# Activation of GPR55 Receptors Exacerbates oxLDL-Induced Lipid Accumulation and Inflammatory Responses, while Reducing Cholesterol Efflux from Human Macrophages

**DOI:** 10.1371/journal.pone.0126839

**Published:** 2015-05-13

**Authors:** Mirko Lanuti, Emanuela Talamonti, Mauro Maccarrone, Valerio Chiurchiù

**Affiliations:** 1 European Center for Brain Research (CERC), IRCCS, Santa Lucia Foundation, Rome, Italy; 2 Center of Integrated Research, Campus Bio-Medico University of Rome, Rome, Italy; University of Padova, ITALY

## Abstract

The G protein-coupled receptor GPR55 has been proposed as a new cannabinoid receptor associated with bone remodelling, nervous system excitability, vascular homeostasis as well as in several pathophysiological conditions including obesity and cancer. However, its physiological role and underlying mechanism remain unclear. In the present work, we demonstrate for the first time its presence in human macrophages and its increased expression in ox-LDL-induced foam cells. In addition, pharmacological activation of GPR55 by its selective agonist O-1602 increased CD36- and SRB-I-mediated lipid accumulation and blocked cholesterol efflux by downregulating ATP-binding cassette (ABC) transporters ABCA1 and ABCG1, as well as enhanced cytokine- and pro-metalloprotease-9 (pro-MMP-9)-induced proinflammatory responses in foam cells. Treatment with cannabidiol, a selective antagonist of GPR55, counteracted these pro-atherogenic and proinflammatory O-1602-mediated effects. Our data suggest that GPR55 could play deleterious role in ox-LDL-induced foam cells and could be a novel pharmacological target to manage atherosclerosis and other related cardiovascular diseases.

## Introduction

Atherosclerosis is a chronic inflammatory disease of large and medium-sized muscular arteries, characterized by endothelial dysfunction and accumulation of lipids, as well as cellular debris and fibrous elements within the intima of the vessel wall. These events result in plaque formation, vascular remodeling, luminal obstruction, ultimately leading to thrombosis [1]. Atherosclerosis is a multifactorial complex disease, where many components of the vascular and immune systems are interconnected and hence determine the degree of severity. The formation of lipid-laden macrophages is one of the earliest events and a hallmark of atherogenesis [1]. The transformation of these macrophages into foam cells is promoted by a dysregulated uptake of cholesterol-rich modified low density lipoproteins (LDL) through several types of scavenger receptors, most notably CD36 and an unbalanced cholesterol efflux through different transporters like ABCA1, ABCG1 and SR-BI [2, 3]. Cholesterol engulfment by macrophages leads to dysregulation of both immune response and cholesterol metabolism of these cells, the former being characterized by up-regulated activation of the NF-kB pathway [4, 5] and, consequently, by enhanced T helper-1 (Th-1) response in the plaque, whereas the latter is mainly associated with activation of the PPAR-γ signalling pathway that increases oxLDL uptake [6, 7]. Even though search of innovative targets for atherosclerosis treatment appears to hold promises, statins remain the more effective pharmacological compounds for clinical management, despite their unwanted side effects and significant residual cardiovascular risk [8]. In the last decade, solid evidence has pointed to the two main receptors that bind to and mediate the biological activity of exogenous (plant-derived or synthetic) cannabinoids and of their endogenous counterparts (endocannabinoids), namely type-1 and type-2 cannabinoid receptors (CB_1_R and CB_2_R), as relevant players in atherosclerosis, endowed with pro-atherogenic [9, 10] and anti-atherogenic effects respectively [11–14]. Yet, the actual role of CB_1_R and CB_2_Rremains controversial, because conflicting results have been reported by independent investigators [15, 16]. In this context, the putative "type 3"cannabinoid receptor GPR55 has attracted growing attention, because it is believed to mediate some of the non-CB_1_R/non-CB_2_R actions of endogenous, plant-derived or synthetic cannabinoids [17]. GPR55 is a G protein-coupled receptor discovered and isolated in 1999 by Sawzdargo and colleagues in human striatum as an orphan receptor; additional studies on its pharmacology and downstream signaling suggested therapeutically relevant scenarios. Indeed, GPR55 expression and activity was documented in a number of (patho)physiological processes, like bone remodelling [18], nervous system excitability [19, 20], neutrophil migration [21, 22], and mast cell degranulation [23]. Intriguingly, the first evidence for a functional role of GPR55 was obtained in the vascular system, where it was shown to regulate systemic vascular resistance and angiogenesis [24, 25]. In the light of GPR55 role in the (patho)physiology of the vascular and immune systems, that are both critically involved in the pathogenesis of atherosclerosis, the aim of our work was to investigate the potential role of this receptor in human macrophage-derived foam cells, in terms of modulation of cholesterol influx/efflux and of inflammatory cytokines production.

## Materials and Methods

### Cell cultures

The human monocytic cell line THP-1 was obtained from the American Type Culture Collection (ATCC, Rockville, MD), and was cultured in Falcon flasks with RPMI-1640 medium supplemented with 10% fetal bovine serum, L-glutamine (2 mM), sodium pyruvate (100 μg/ml), penicillin (100 U/ml) and streptomycin (100 μg/ml). Cells were cultured at 37°C in a humidified 5% CO_2_ atmosphere. THP-1 monocytes were differentiated into macrophages in the presence of 100 nM phorbol 12-myristate 13-acetate (PMA, Sigma Aldrich, Italy) for 72h, as reported [26, 27, 28]. All cell pre-treatments and treatments were performed in absence of serum.

### LDL oxidation, foam cell formation and treatments

LDL (Sigma Aldrich) were reconstituted in deionized water (1 mg LDL protein/ml), and were oxidized at 37°C by incubation with 5 μM Cu_2_SO_4_ for 24h. Lipoprotein oxidation was assessed by changes in absorbance at 234 nm (due to conjugated dienes), and Cu^2+^ ions were removed by extensive dialysis. PMA-differentiated THP-1 macrophages were incubated with 100 μg/ml of oxLDL for 18h, in order to allow lipoproteins uptake, and hence conversion of macrophages into foam cells as reported [11, 29], without affecting cell viability. Ox-LDL-derived foam cells were treated with GPR55 selective agonist 5-Methyl-4-3-methyl-6-(1-cyclohexen-1-yl)-1,3-benzendiol [O-1602] alone or in combination with its antagonist cannabidiol [CBD] for 24 hours.

### qRT-PCR

RNA was extracted from human control or lipid-laden THP-1 macrophages, using the RNeasy extraction kit (Qiagen, Crawley, UK), as suggested by the manufacturer. Total RNA was used for reverse transcriptase (RT) reaction, performed by means of the iScript TM cDNA Synthesis kit (Bio-Rad, Hercules, CA, USA), as reported. The following program was used for the quantitative RT-PCR: 25°C for 10 min, 42°C for 50 min, 85°C for 5 min, then after addition of 0.1 unit/ml of *Escherichia coli* RNase H, the product was incubated at 37°C for 20 min. The target transcripts were amplified by means of an ABI PRISM 7700 sequence detector system (Applied Biosystems, Foster City, CA), using the following specific human primers for: GPR55 F1 (5’-CACCGGCCTTCCAGGGTCCA-3’) and GPR55 R1 (5’-CCCGGGAGATCGTGGTGTCCT-3’); NFAT-C1 F1 (5’-TTCCCGCCAACGGTAACGCC-3’) and NFAT-C1 R1 (5’-AAAAGCACCCCACGCGCTCA-3’); NFAT-C2 F1 (5’-CCGAGTCCCCCTCCTGCCTC-3’) and NFAT-C2 R1 (5’-GCTGGGGCTCAGGCTCTTGC-3’); NFAT5 F1 (5’- AGCAGAGGCCGGGGGTCAAA-3’) and NFAT5 R1 (5’-GGTGCTTTGGCACTGTCGGC-3’). β-Actin was used as housekeeping gene for quantity normalization. One μl of the first strand of cDNA product was used for amplification (in triplicate) in a 25 μl reaction solution, containing 12.5 μl of Platinum SYBRGreen qPCR Super-Mix UDG (Invitrogen) and 10 pmol of each primer. The following PCR program was used: 95°C for 10 min, 40 amplification cycles at 95°C for 30 s, 56°C for 30 s, and 72°C for 30 s, as reported [30].

### Immunoblotting

Control THP-1 macrophages or THP-1-derived foam cells, seeded at 3x10^6^ cells/well, were washed 3 times with cold phosphate buffer saline (PBS), incubated for 3 min with accutase and collected for cell lysis. Then, cells were lysed with RIPA buffer, and protein expression assessed by Western blotting, as reported [30]. Cell homogenates were subjected to 10% SDS-PAGE (40 μg/lane) under reducing conditions, then gels were electroblotted onto 0.45-mm nitrocellulose filters and were immunoreacted with anti-GPR55 (1:200, Cayman), anti-ABCA1 (1:1000, Abcam), anti-ABCG1 (1:1000, Abcam), anti-SRBI (1:1000, Abcam) or with anti-β-actin monoclonal antibody (1:20000, Sigma Aldrich). Proteins were detected by enhanced chemiluminescence (ECL; Amersham Pharmacia Biotech) and by exposure to X-ray film (Hyper ECL; Amersham Pharmacia Biotech), quantitating band intensity by densitometry in a ChemiImager 4400 apparatus (Alpha Innotech, San Leandro, CA).

### Confocal microscopy

Confocal microscopy was used to evaluate the cellular content of lipid droplets through Nile red staining, as well as to localize the expression of GPR55. Briefly, control THP-1 macrophages or THP-1-derived foam cells (10x10^5^ cells/well) were fixed on coverglasses, and permeabilized either with 0.1% Triton-X100 or 0.1% saponin, prior to Nile red staining or incubation with anti-GPR55 polyclonal antibody (1:100, Cayman), respectively. Lipid droplets and GPR55 receptor expression were visualized by means of a Zeiss LSM700 Confocal microscope, and were analyzed through ImageJ software (National Institutes of Health, Bethesda, MD, USA), as reported [31].

### Zymography

Gelatinase activity was measured in conditioned medium derived from cells subjected to the different experimental conditions. Samples (15μl) were mixed at room temperature with 35μl of double-strength non-reducing sample buffer and then subjected to electrophoresis at room temperature into a 8% (w/v) polyacrylamide gel containing 10% SDS and of gelatin from rat tail collagen (1mg/ml). After electrophoresis, SDS was removed from gels by 3 washes for 30 minutes each with aqueous solution of 2.5% (v/v) Triton X-100. To activate the metalloproteinases the gel was then incubated overnight at 37°C in a buffer containing 50 nM Tris HCl (pH 7.6), 10 mM CaCl_2_, 0.02% NaN_3_. The gel was then stained for 15 minutes with aqueous solution of 0.0025% Comassie Brilliant Blue R-250, 20% Glacial Acetic Acid, 2.3% Methanol. To unstain the excess of Blue Brilliant Comassie the gel was incubated with aqueous solution of 10% Glacial acetic Acid, 40% Methanol. Zones of lysis appeared as clear bands against the blue-stained background, and these bands were analyzed by means of ImageJ software.

### Flow cytometry

Control THP-1 and foam cells, primed overnight with LPS and untreated or incubated with GPR55 agonist or antagonist (alone or in combination), were washed 3 times with PBS, collected by means of accutase and then stained with FITC-conjugated anti-CD36 specific antibody. Intracellular staining for tumor necrosis factor α (TNF-α) and IL-10 was performed prior to cytokine inhibition, by adding 1 μg/ml brefeldine A (Sigma) 4h before cell collection. Cells were then fixed with 4% formaldehyde (Sigma) for 10 min at room temperature, and were stained in saponine 0.5% with anti-TNF-α APC and anti-IL10 PE. Flow cytometry as also used to assess GPR55 expression by staining macrophages and foam cells with GPR55 antibody both at cell surface or intracellulary upon cell permeabilization with saponine 4% formaldehyde and 0.5% saponine. Flow cytometry analysis was performed on a FACSCanto Flow Cytometer (Beckton Dickinson, NJ, USA), as reported [31].

### Statistical analysis

Data are expressed as the mean ± SD. Statistical analysis of data was performed using the Student’s t-test or parametric one-way ANOVA followed by Tukey post-hoc test on Prism GraphPAD 5.0. Differences in data were considered to be statistically significant for P < 0.05.

## Results

### GPR55 expression in human macrophages and foam cells

**Fig 1:**
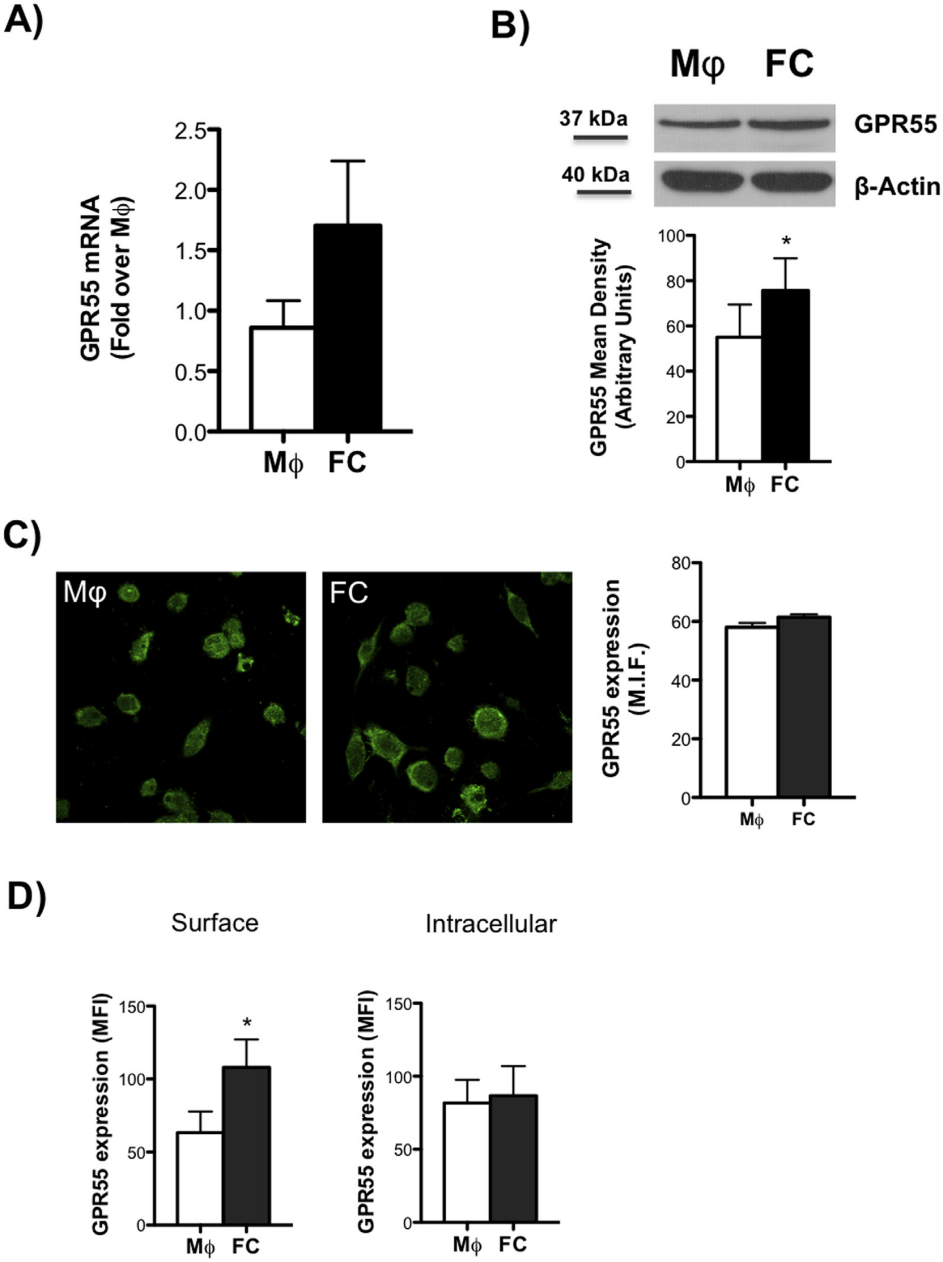
mRNA and protein analysis of GPR55 in human macrophages and foam cells. Human THP-1 macrophages (MΦ) were left untreated or were treated for 18 h with 100 μg/ml oxLDL to generate foam cells (FC). (A) mRNA content of GPR55 was analyzed by qRT-PCR. Data are shown as mean (±SD) of five independent experiments, each in duplicate. (B) Western blot analysis of GPR55, with the expected molecular mass of each protein shown on the left-hand side. Data are expressed in comparison with β-actin, and are the mean (± SD) of five independent experiments, each performed in duplicate. *p<0.05 *versus* MΦ. (C) Immunofluorescence of GPR55 was performed by confocal laser-scanning microscopy, and data are shown as pictures taken with a 63 objective (numerical aperture 1⁄4 1.4), and as densitometric analysis (mean ±SD) of at least six independent experiments. (D) GPR55 expression was assessed by flow cytometry upon staining cells with anti-GPR55 FITC either at cell surface or intracellularly upon cell permeabilization. Data are reported as mean fluorescence intensity (M.I.F.), and are representative of five independent experiments. *p<0.05 *versus* MΦ.

The expression of GPR55 receptor was documented at transcriptional and translational levels in both human THP-1 macrophages (MΦ) and macrophage-derived lipid laden foam cells (FC). We firstly evaluated the mRNA transcript for GPR55 by qRT-PCR, and found that it was upregulated (yet not significantly) in FC compared to MΦ (Fig 1A). Then, we further investigated protein expression by immunoblotting, confocal microscopy and flow cytometry. We could document a significant increase of GPR55 total protein content in FC compared to MΦ by immunoblotting (Fig 1B), though no significant changes could be detected by confocal microscopy (Fig 1C). Interestingly, the confocal images show diffuse cytoplasmic receptor staining, whereas in foam cells the positive signal is more localized at the plasma membrane. However, analysis by flow cytometry showed a significant upregulation of this receptor in FC at cell surface but not intracellularly (Fig 1D), rather suggesting a change in subcellular receptor localization than increased expression upon oxLDL-induced FC formation. GPR55 antibody specificity was tested in non-expressing HEK293 cell line, as previously reported [32].

### Effects of GPR55 activation on CD36-induced lipid-droplets accumulation and cholesterol transporters expression in human foam cells

**Fig 2:**
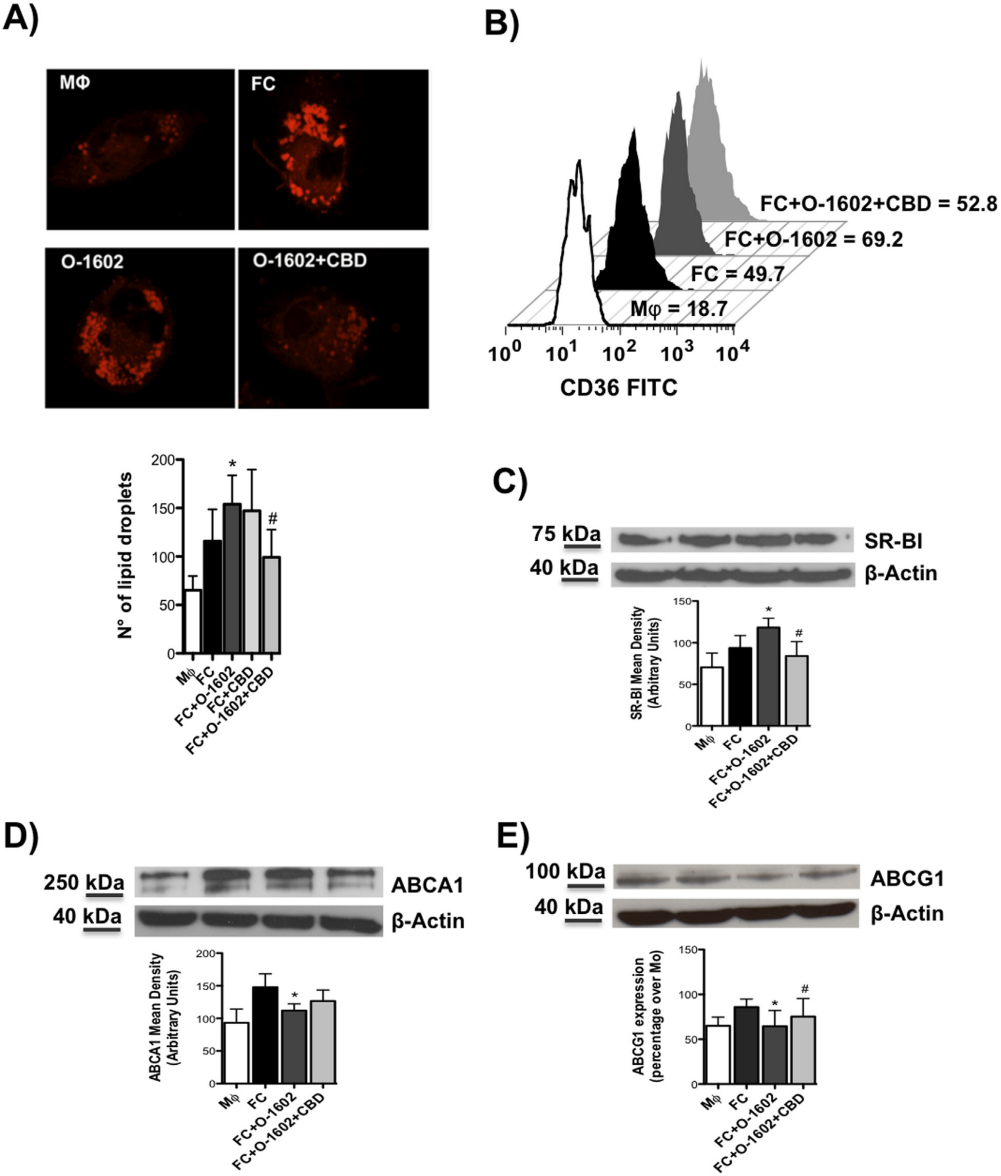
Regulation of lipid-droplets accumulation and cholesterol transporters in human macrophages and foam cells. Human THP-1 macrophages (MΦ) were left untreated or were treated for 18 h with 100 μg/ ml oxLDL to generate foam cells (FC), that were then treated for further 24 h with selective GPR55 agonist O-1602, and were pretreated or not with the CBD antagonist. (A) Nile red-stained lipid droplets were analyzed by confocal laser-scanning microscopy, and data are shown as numbers of lipid-droplets per cell (mean ±SD) of at least six independent experiments. Representative images of MΦ and FC, as well as of FC treated with 10nM O-1602or 0.5 μM CBD are shown in the panel. *p<0.05*versus* MΦ; #p<0.05 *versus*FC+O-1602. (B) Effect of GPR55 activation on CD36 expression in human macrophages and foam cells. The latter cells were pretreated with the CBD antagonist, and then were treated with the O-1602 agonist. CD36 expression was assessed by flow cytometry upon staining cells with anti-CD36 FITC. Data are reported as mean fluorescence intensity, and are representative of four independent experiments. Western blot analysis of SR-BI (C), ABCA1 (D), ABCG1 (E), with the expected molecular mass of each protein shown on the left-hand side. Data are expressed in comparison with β-actin, and are the mean (±SD) of five independent experiments, each performed in duplicate. *p<0.05 *versus* MΦ; #p<0.05 *versus* FC+O-1602.

The re-localization of GPR55 in FC compared to MΦ at cell surface led us to interrogate whether its activation had any effect on CD36-mediated uptake and subsequent intracellular accumulation of oxLDL in lipid-droplets, as well as on modulation of cholesterol efflux. To this aim, we treated FC with the selective GPR55 synthetic agonist O-1602. Since previous data by our group and others reported low nanomolar effects for O-1602 in vitro [32–34], we tested several concentrations of this compound in this foam cell in vitro model, and we found that the lowest dose with an efficacious effect on lipid-droplets accumulation was 10 nM (S1 Fig), and this concentration was used for all subsequent experiments. Indeed, microscopy panels of Nile red staining of lipid droplets show a significant increase in the content of intracellular ox-LDL in MΦ treated with O-1602 10nM compared to FC (Fig 2A). To confirm that this effect was likely mediated by GPR55, we also pre-treated FC with the selective receptor antagonist CBD (500 nM), and we found that the blockade of GPR55 potently reduced the intracellular content of lipid droplets. The treatment with CBD alone did not exert any significant effect on intracellular lipid droplet accumulation (Fig 2A). Furthermore, other agonists of GPR55 were tested, i.e. the synthetic Abnormal cannabidiol (Abn-CBD) and the two endogenous ligands Lysophosphatidylinositol (LPI) and Palmitoylethanolamide (PEA), and we found that all of them induced an increase in lipid-droplets accumulation (S1 Fig), with LPI being the most efficacious since it is believed to be the actual endogenous ligand for GPR55 in vivo, whereas Abn-CBD and PEA also bind to GPR18 and PPAR, respectively [17].

The uptake of ox-LDL is mediated by scavenger receptors, especially by CD36 and SR-BI, therefore we further ascertained whether the pharmacological modulation of GPR55 could affect the level of expression of these two main scavenger receptors. Fig 2B and 2C show that oxLDL-induced FC markedly increase protein expression of both CD36 and SR-BI, that was even higher after treatment with O-1602. Additionally, selective blockade of GPR55 with CBD significantly reduced lipid droplets accumulation in FC. Next, we further extended our investigation to the possible role of GPR55 on protein expression of the principal transporters that regulate cholesterol efflux in MΦ, i.e. ATP-binding cassettes ABCA1 and ABCG1. We observed a significant down-regulation of both ABCA1 and ABCG1 transporters upon GRP55 activation, an effect that was counteracted by CBD, but only in the case of ABCG1 and not of ABCA1 (Fig 2D and 2E).

### Immunomodulatory role of GPR55 activation in human foam cells

**Fig 3:**
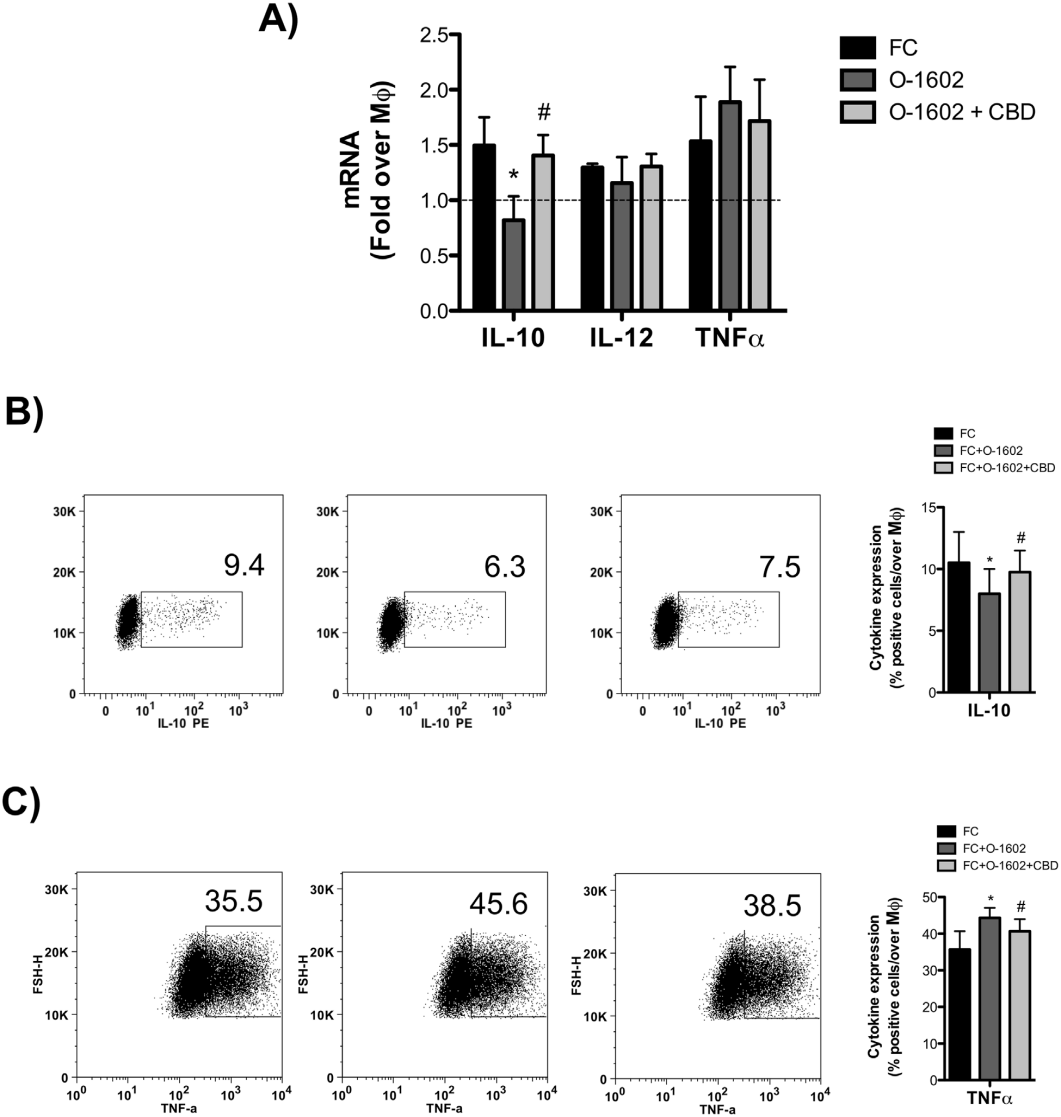
Effect of GPR55 activation on cytokines production and release. THP-1 macrophages (MΦ) were left untreated or were treated for 18 h with 100 mg/ml oxLDL to generate foam cells (FC). FC were treated for 24h with O-1602, and pretreated or not with CDB. (A) mRNA content of IL-10, IL-12, TNF-α were analyzed by qRT-PCR. Data are shown as mean (±SD) of six independent experiments, each in duplicate. *p<0.05 *versus* FC; #p<0.05 *versus* FC+O-1602. Levels of intracellular cytokines production were assessed by flow cytometry (as detailed in Materials and Methods), upon intracellular staining with anti-TNF-α (B) and IL-10 (C) antibodies. Data are means (±SD) of four independent experiments. *p<0.05 *versus* FC; #p<0.05 *versus* FC+O-1602.

**Fig 4:**
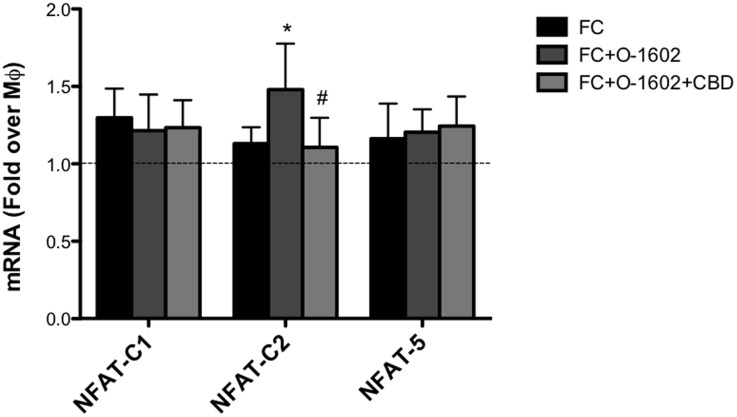
Effect of GPR55 activation on MMP-9 activity. Human THP-1 macrophages (MΦ) were left untreated or were treated for 18 h with 100 μg/ml oxLDL to generate foam cells (FC). FC were treated for 24h with O-1602, and pretreated or not with CDB. After the treatments, surnatants were collected and gelatinase activity of MMP-9 was measured in conditioned media subjected to zymography. Gelatinase activity is expressed as area (mm^2^) exposed to MMP-9 activity. Data are shown as mean ± SD of five independent experiments. *p<0.05 *versus* FC; #p<0.05 *versus* FC+O-1602.

**Fig 5:**
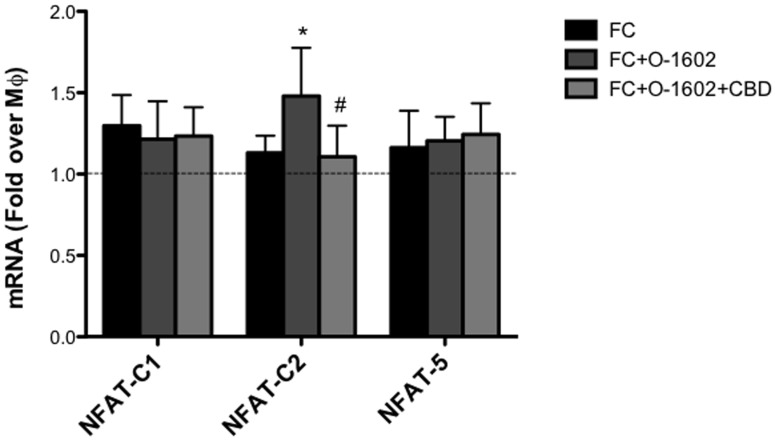
Effect of GPR55 activation on NFAT transcription factor members. Human THP-1 macrophages (MΦ) were left untreated or were treated for 18 h with 100 μg/ml oxLDL to generate foam cells (FC). FC were treated for 24h with O-1602, and pre-treated or not with CDB. mRNA analysis of NFAT members was performed by qRT-PCR. Data are shown as mean ± SD of three independent experiments, each in duplicate. *p<0.05 *versus* FC; #p<0.05 *versus* FC+O-1602.

Besides bearing a disrupted cholesterol homeostasis, oxLDL-induced FC are also characterized by an overt proinflammatory phenotype. Thus, we next evaluated the possible impact of GPR55 activation on the production of inflammatory cytokines. At the transcriptional level, FC expressed higher levels of IL-12, TNF-α and IL-10 compared to MΦ (Fig 3A). When cells were treated with O-1602, TNF-α mRNA levels were further increased, whereas those of IL-10 significantly decreased. Instead, IL-12 transcription was hardly affected by GPR55 activation. Next, protein expression of IL-10 and TNF-α was evaluated by means of flow cytometry. Intracellular cytokine staining revealed a ∼1.4-fold decrease in IL-10 production (Fig 3B), and a parallel ∼1.3-fold increase in TNF-α (Fig 3C) in FC treated with O-1602, and these effects were significantly reverted by CBD, further supporting the proinflammatory nature of GPR55 in these cells. OxLDL-activated FC also produce extracellular matrix-degrading metalloproteinases (MMP), which are involved in atherosclerotic plaque instability. Thus, we assessed the possible effect of GPR55 activation on MMP-9 production by means of zymography. Interestingly, we found that O-1602-treated FC supernatants had higher MMP-9 activity than those of untreated FC, and that such an enzyme activity was significantly reverted by CBD (Fig 4). Since GPR55 downstream signaling engages Rho, which then stimulates intracellular calcium release and nuclear translocation of the nuclear factor of activated T-cells (NFAT) [35], we sought to investigate whether GRP55 could modulate gene expression of the main members of NFAT in FC. To this aim, we analyzed mRNA levels of NFAT-c1, NFAT-c2 and NFAT5, which are all largely expressed in macrophages [36], upon GPR55 activation or blockade. qRT-PCR analysis showed that only NFAT-c2 mRNA levels were upregulated by GPR55 activation, an effect that was specifically blunted by receptor antagonism with CBD (Fig 5). No changes in NFAT-c1 and NFAT5 expression were observed upon GPR55 activation, suggesting that the proinflammatory effects of GPR55 shown in this study could be mediated by the NFAT-c2 transcription factor.

## Discussion

Over the last few years, the basic mechanisms underlying the pathophysiology of atherosclerosis have considerably evolved, and details of the immunopathology of the disease have significantly progressed. Inflammation is a key hallmark of atherosclerosis, and a great body of evidence shows that a complex crosstalk between innate and adaptive immune cells can ultimately affect the severity of atherosclerosis-related inflammatory events [37]. The starting event is the accumulation of MΦ-derived FC underneath the endothelial wall, where they play a crucial role in onset and progression of the atherosclerotic plaque, due to dysregulated clearance of modified LDL and subsequent triggering of an inflammatory cascade. Accumulated evidence has highlighted a regulatory role of cannabinoids on cardiometabolic diseases such as dyslipidemia, obesity and diabetes [38]. Intriguingly, the effects mediated by cannabinoids *via* their receptors in atherosclerosis are opposite: activation of CB_1_R is closely associated with multiple cardiometabolic risk factors [38, 39], whereas activation of CB_2_R in immune cells shows protective and anti-inflammatory effects [11,15]. Recently, another putative "type 3"cannabinoid receptor, GPR55, has been described, and has been supposed to play a physiological role in pain signaling, control of vascular tone, and inflammation. In the present investigation, we document the expression of GPR55 in human MΦ and FC, showing that such a receptor might bear a pro-atherogenic and proinflammatory activity.

The crucial event in FC formation is a dysregulated uptake of oxLDL, which leads to a massive intracellular accumulation of cholesterol esters followed by activation of distinct proinflammatory pathways. Our findings indicate that the selective activation of GPR55 might have an impact on the regulation of oxLDL accumulation and on cholesterol influx/efflux through transporters. Indeed, by up-regulating the expression of CD36 and SR-BI scavenger receptors, and by concomitantly down-regulating the expression of ABCG1 and (to a lower extent) ABCA1 transporters, GPR55 is more likely to play a pro-atherogenic role. Under physiological conditions, oxysterols and cholesterol esters within MΦ are sensed by the nuclear factors Liver X receptor (LXR) and Retinoic X receptor (RXR), leading to ABCA1 and ABCG1-mediated cholesterol efflux [40, 41]. In FC, the unconstrained accumulation of modified LDL by scavenger receptors not only disrupts the pathways that govern cholesterol homeostasis but also activates pro-inflammatory NF-kB-mediated signaling, thus increasing the release of proinflammatory mediators and ultimately enhancing the expression of scavenger receptors [1]. Our results suggest that GPR55 might interfere with these pathways, perhaps by increasing oxLDL intracellular accumulation and/or decreasing cholesterol efflux, along with favoring the production of pro-inflammatory cytokines at the expenses of the anti-inflammatory ones. The GPR55-induced increase of proinflammatory TNF-α, which plays a pivotal role in atherogenesis and in orchestrating the production of a pro-inflammatory cytokine cascade [42–45], along with the concomitant reduction of IL-10, which is highly atheroprotective [46], support the presumed proinflammatory nature of GPR55. Incidentally, very recently the anti-atherogenic effects of IL-10 have been demonstrated to be mediated by enhanced ABCA1- and ABCG1-mediated cholesterol efflux [47]. The noxious regulatory loop promoted by GPR55 in FC was further supported by receptor ability to increase MMP-9 activation. Interestingly, the regulation of MMP-9 expression has been associated with the signaling pathways triggered by Rho GTPases [48], that are among the major executioners engaged by GPR55 and that play a role in actin cytoskeleton reorganization [49]. In this context, our findings seem to suggest that GPR55 may be involved in plaque stability, thus affecting rupture of the fibrous cap and eventually inducing thrombus formation. When compared to the "classical" CB_1_ and CB_2_ receptors, the effects exerted by GPR55 in atherosclerosis are similar to those of the former and opposite to those of the latter receptor. Of note, several GPR55 selective ligands are also agonists or inverse agonists/antagonists of CB_1_R but not of CB_2_R [50, 51], suggesting possible cross-talks or synergism between these two atherogenic and pro-inflammatory receptors.

Although this is the first evidence reporting the involvement of GPR55 in macrophage-to-foam cells formation with an impact on atherosclerosis, our findings seem to be supported by recent reports showing that GPR55 does regulate other cardiovascular-related phenomena, including food intake and adiposity [52–54], glucose-stimulated insulin secretion [55], and platelet and endothelial function [56, 57]. Taken together, our study suggests that GPR55 could be a novel pharmacological target to combat atherosclerosis and other related cardiovascular diseases.

## Supporting Information

S1 Fig Lipid-droplets accumulation in human macrophages and foam cells by different concentrations of O-1602 and other agonists of GPR55. Human THP-1 macrophages (MΦ) were left untreated or were treated for 18 h with 100 μg/ ml oxLDL to generate foam cells (FC), that were then treated for further 24 h with selective GPR55 agonist O-1602 at different concentrations (1–100nM) (**A**) or with GPR55 agonists Abn-CBD, LPI and PEA (**B**). Nile red-stained lipid droplets were analyzed by confocal laser-scanning microscopy, and data are shown as numbers of lipid-droplets per cell (mean ±SD) of at least four independent experiments. *p<0.05 *versus* FC; **p<0.01 *versus* FC.
(TIFF)
