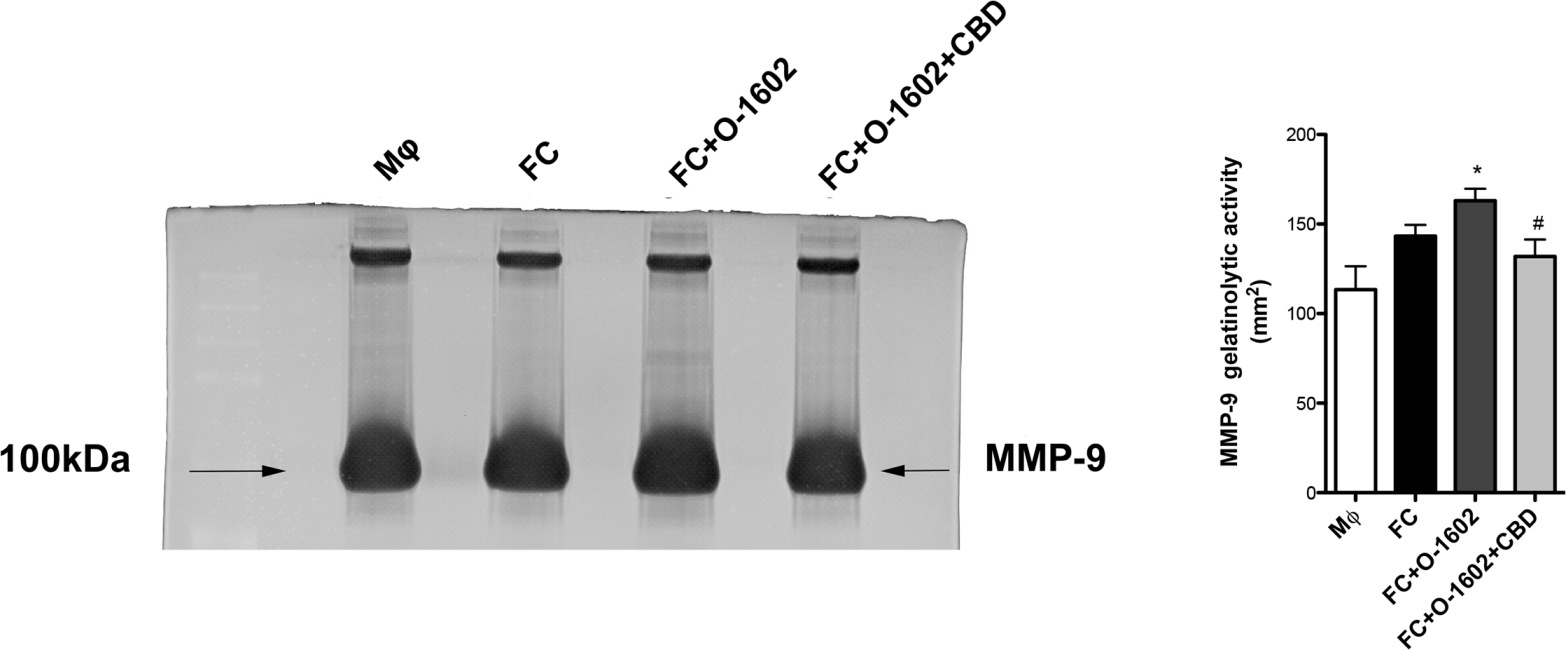# Correction: Activation of GPR55 Receptors Exacerbates oxLDL-Induced Lipid Accumulation and Inflammatory Responses, while Reducing Cholesterol Efflux from Human Macrophages

**DOI:** 10.1371/journal.pone.0131850

**Published:** 2015-06-25

**Authors:** Mirko Lanuti, Emanuela Talamonti, Mauro Maccarrone, Valerio Chiurchiù

Fig 4 is incorrect. The correct Fig 4 is available here.

**Figure:**