# Supplementary figures and images for: Activation of GPR55 Receptors Exacerbates oxLDL-Induced Lipid Accumulation and Inflammatory Responses, while Reducing Cholesterol Efflux from Human Macrophages
Source: PLoS One. 2015 May 13;10(5):e0126839. doi: 10.1371/journal.pone.0126839 (PMC4430319; doi:10.1371/journal.pone.0126839)

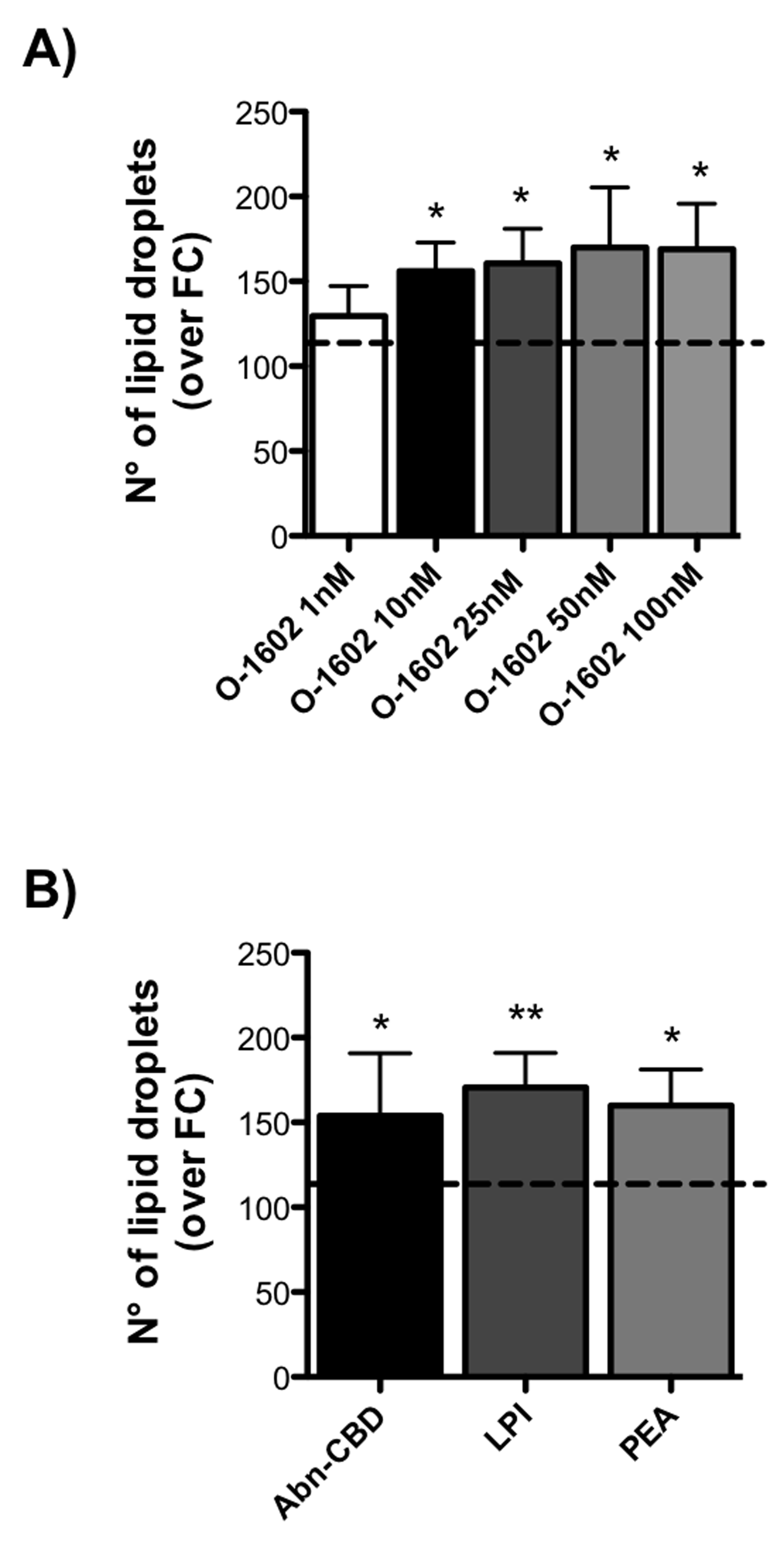

Supplement: S1 Fig — Human THP-1 macrophages (MΦ) were left untreated or were treated for 18 h with 100 μg/ ml oxLDL to generate foam cells (FC), that were then treated for further 24 h with selective GPR55 agonist O-1602 at different concentrations (1–100nM) (A) or with GPR55 agonists Abn-CBD, LPI and PEA (B). Nile red-stained lipid droplets were analyzed by confocal laser-scanning microscopy, and data are shown as numbers of lipid-droplets per cell (mean ±SD) of at least four independent experiments. *p<0.05 versus FC; **p<0.01 versus FC. (TIFF) [file pone.0126839.s001.tiff]
